# A predictable cascade of junctional failure following progressive long-segment fusion: an 11-year case analysis

**DOI:** 10.1093/jscr/rjag429

**Published:** 2026-06-03

**Authors:** Majd Abouassi, Jörg Silbermann, Wahab Moustafa

**Affiliations:** Department of Spine Surgery and Neurotraumatology, SRH Waldklinikum Gera, Gera 07548, Germany; Department of Spine Surgery and Neurotraumatology, SRH Waldklinikum Gera, Gera 07548, Germany; Department of Spine Surgery and Neurotraumatology, SRH Waldklinikum Gera, Gera 07548, Germany

**Keywords:** adjacent segment degeneration, long-segment spinal fusion, hardware failure, pseudarthrosis, revision spine surgery, sagittal imbalance

## Abstract

Instrumented spinal fusion is a common treatment for degenerative spinal disease but may lead to progressive mechanical and biological complications, especially in long multilevel constructs. We report the case of a 62-year-old woman who developed serial adjacent segment degeneration, pseudarthrosis, implant failure, vertebral fractures, and progressive deformity over 11 years following posterior lumbar fusion. Multiple revision surgeries with stepwise cranial extension to the upper thoracic spine were required. Despite advanced fixation techniques—including multi-rod constructs, iliac fixation, and CT-navigated instrumentation—recurrent mechanical failure occurred. The final presentation involved an unstable thoracic fracture with impending spinal cord injury, necessitating urgent revision with vertebral body replacement and cranial extension into the cervical region. This case highlights the cumulative biomechanical and biological burden of long-segment fusion and underscores the importance of careful patient selection, bone health optimization, and long-term follow-up.

## Introduction

Posterior spinal fusion with pedicle screw instrumentation is widely used for degenerative spinal disease. Although short-segment fusion often yields durable symptom relief, long multilevel constructs are associated with increased mechanical stress, higher revision rates, and a greater incidence of adjacent segment degeneration (ASD) [1, 2], particularly in elderly and osteoporotic patients. While most reports describe isolated complications, longitudinal documentation of serial adjacent segment failure with recurrent hardware complications and progressive neurological deterioration over more than a decade is rare. This case illustrates the cumulative impact of repeated mechanical and biological failure mechanisms over an 11-year course.

## Case presentation

**Figure 1:**
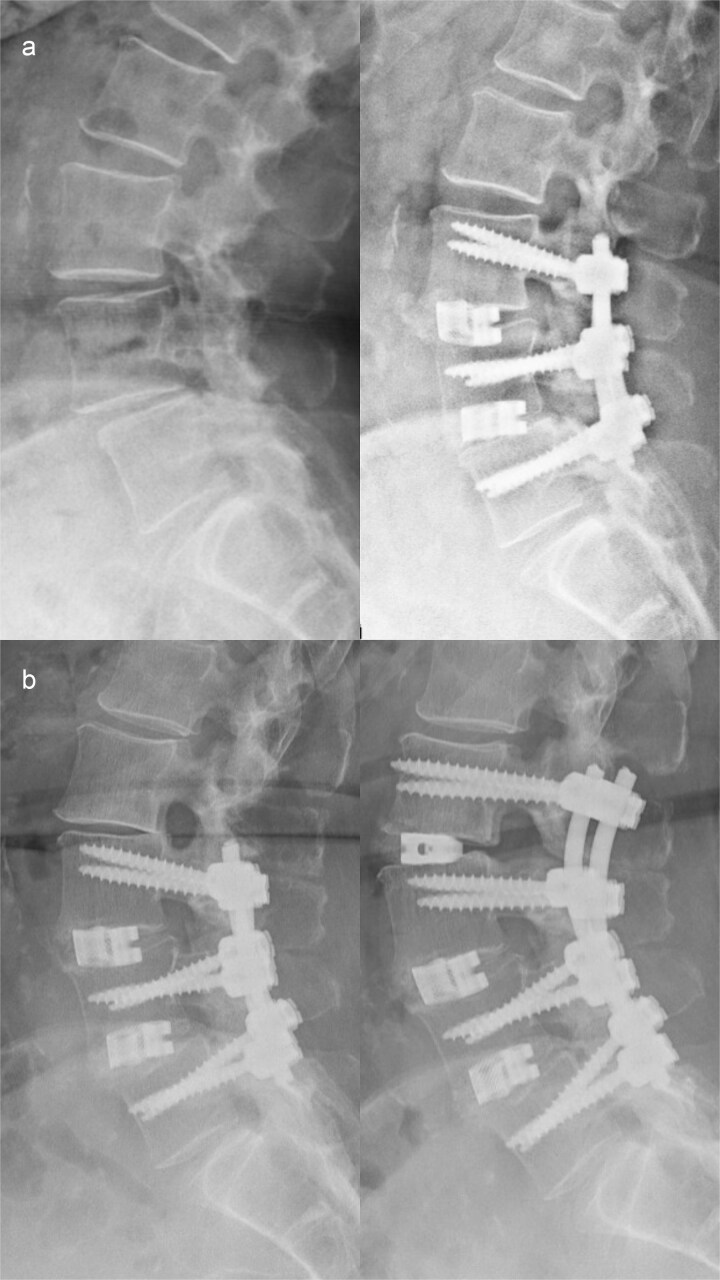
(a) Preoperative imaging demonstrating segmental instability at L3–L5 (left) and postoperative imaging following posterior lumbar interbody fusion (PLIF) from L3 to L5 (right). (b) Preoperative imaging showing cranial adjacent segment degeneration at L2/3 above the existing fusion (left) and postoperative imaging after extension of the spondylodesis with PLIF at L2/3 (right).

A 62-year-old woman presented in 2014 with low back pain, neurogenic claudication, and right-sided radiculopathy. Radiographs demonstrated lumbar spinal canal stenosis with segmental instability at L3–L5 without relevant global sagittal imbalance. Because conservative treatment failed and instability was considered symptomatic, posterior lumbar interbody fusion (PLIF) from L3–L5 was performed (Fig. 1a).

In 2018, symptomatic degeneration occurred at L2/3 cranial to the construct, consistent with proximal junctional overload. The fusion was extended with PLIF at L2/3 (Fig. 1b).

**Figure 2:**
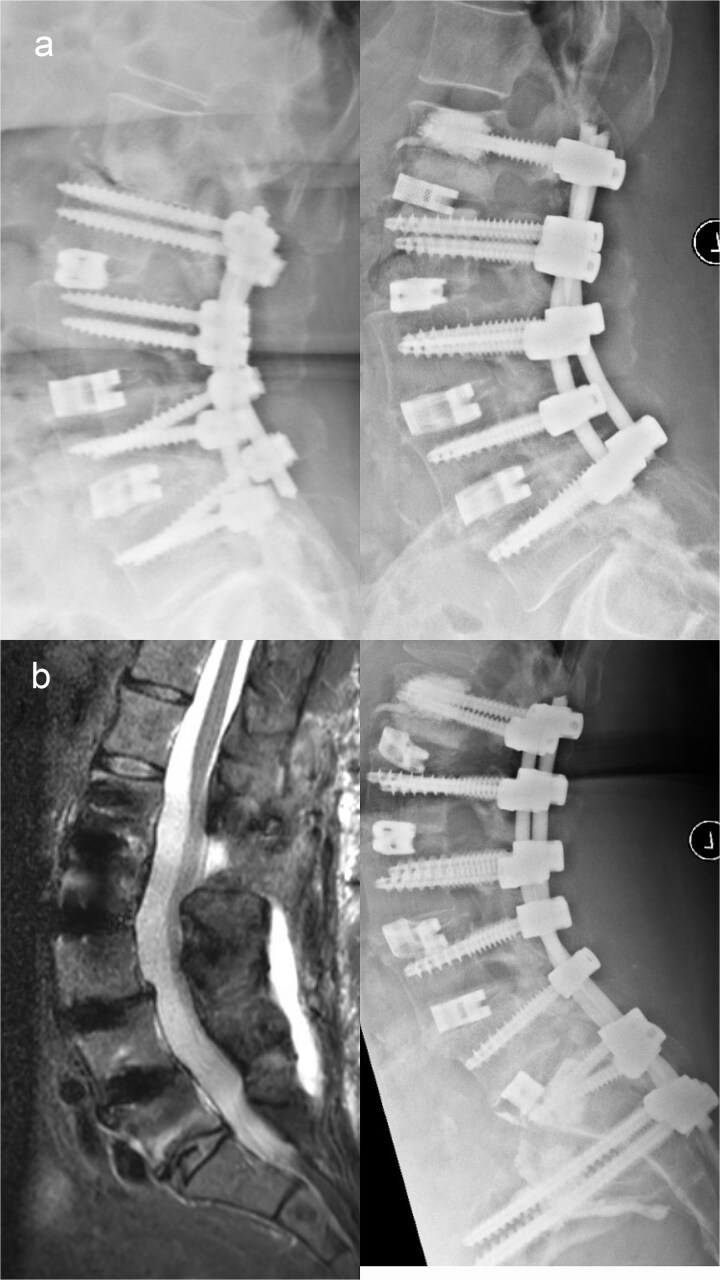
(a) Preoperative imaging demonstrating pronounced cranial adjacent segment degeneration at L1/2 with severe spinal canal stenosis and foraminal narrowing (left), and postoperative imaging following extension of the fusion to L1–L5 with left-sided TLIF at L1/2 (right). (b) Preoperative magnetic resonance imaging showing a fresh inferior endplate fracture of L5 (left), and postoperative imaging after extension of the spondylodesis to the ilium (right).

In early 2020, progressive neurological deficits developed due to collapse and stenosis at L1/2, representing ongoing proximal junctional failure (PJF). Fusion was extended to L1 with left-sided TLIF at L1/2 (Fig. 2a).

Shortly thereafter, acute L5 radiculopathy occurred. Imaging revealed screw loosening and inferior endplate fracture at L5/S1, interpreted as distal junctional failure from increasing cantilever forces; lumbopelvic fixation was performed (Fig. 2b).

**Figure 3:**
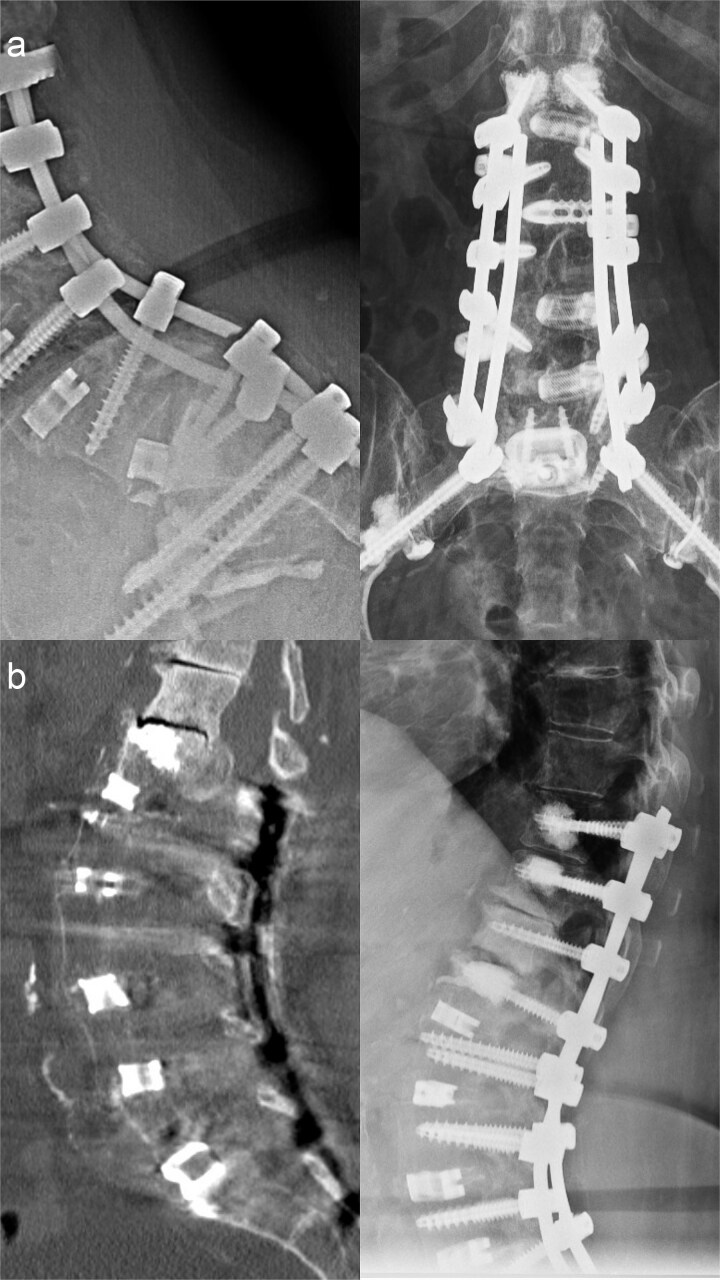
(a) Preoperative imaging demonstrating bilateral rod fracture following fusion from L1 to the ilium (left), and postoperative imaging after posterior revision with rod exchange, conversion to a four-rod construct across the lumbosacral junction, and subsequent anterior revision with cage exchange and ventral re-spondylodesis (right). (b) Preoperative imaging showing cranial adjacent segment degeneration at T12/L1 with an ossified intraspinal mass (left), and postoperative imaging following extension of the fusion to T10 (right).

By late 2021, bilateral rod fracture and lumbosacral pseudarthrosis developed, indicating mechanical overload with insufficient fusion. Revision with four-rod stabilization and staged anterior revision with cage exchange and ventral re-spondylodesis via a Pfannenstiel approach was performed (Fig. 3a).

In May 2025, the patient experienced progressive lumbar pain accompanied by L1–L2 radicular symptoms. Imaging revealed degeneration at T12/L1 associated with an ossified intraspinal mass with progressive sagittal imbalance, reflecting cranial migration of the stress junction. Proximal extension of the fusion to T10 was required with pedicle subtraction osteotomy at L1 (Fig. 3b).

**Figure 4:**
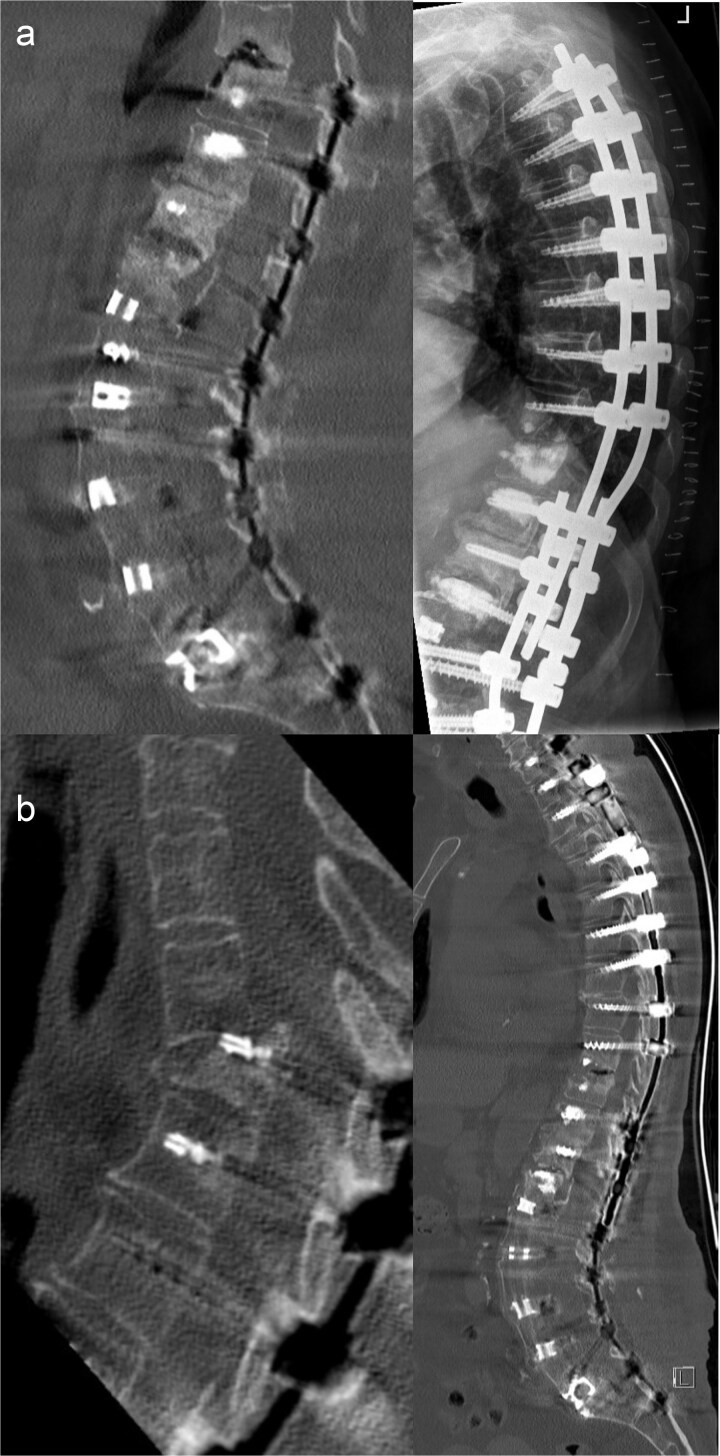
(a) Preoperative magnetic resonance imaging demonstrating an adjacent segment fracture at T9 (left), and postoperative imaging following CT-navigated posterior instrumentation from T3 to T8, including removal of the T10 screw pair and integration of the new construct into the existing instrumentation using cross-connectors (right). (b) Preoperative imaging showing a highly unstable T3 vertebral body fracture (left), and postoperative imaging following urgent CT-navigated posterior fusion from C7 to T2 (right).

By August 2025, pain intensified and progressive sagittal decompensation occurred. Imaging demonstrated a fracture at T9 and screw dislocation at T10, consistent with structural PJF due to reciprocal thoracic kyphosis above the long construct. Stabilization from T3 to T8, including removal of the T10 screw pair was performed and integrated into the existing instrumentation (Fig. 4a).

In October 2025, the patient required emergency admission because of acute thoracic pain and rapidly progressive neurological deterioration. Imaging showed a highly unstable atraumatic fracture of T3 with impending spinal cord injury, representing catastrophic PJF at the cervicothoracic transition. Urgent posterior fusion from C7 to T2 with vertebral body replacement at T3 was performed incorporating end-to-end rod connection and left-sided double-rod augmentation (Fig. 4b).

Postoperatively, transient delirium, hemodynamic instability, and suspected transient ischemic attacks occurred, reflecting systemic frailty after repeated major spinal reconstruction; all resolved with supportive medical management.

## Discussion

This case demonstrates the cumulative biomechanical and biological burden associated with long-segment spinal fusion over more than a decade. While spinal fusion is an established treatment for degenerative disease, increasing construct length substantially raises the risk of late complications, particularly ASD, pseudarthrosis, and junctional failure.

ASD is among the most frequently reported long-term sequelae after lumbar fusion. In a landmark longitudinal study, Ghiselli *et al*. reported a progressive increase in radiographic and symptomatic ASD over long-term follow-up [1]. The risk of ASD increases with construct length and altered biomechanics, as fusion transfers motion and stress to adjacent mobile segments [2]. In the present case, adjacent degeneration progressed stepwise from the lumbar spine into the thoracolumbar and upper thoracic regions, illustrating the serial nature of this phenomenon in long constructs.

Pseudarthrosis represented a major driver of mechanical instability and repeated revision surgery in this patient. Failure of fusion has been shown to correlate with persistent pain, implant loosening, and rod fracture. Comparative outcome studies indicate that patients undergoing revision surgery for lumbar pseudarthrosis often experience inferior clinical outcomes compared with those revised for adjacent segment disease [3]. Advanced age, multilevel fusion, and compromised bone quality are recognized risk factors for nonunion, all of which were present in this case [4].

Mechanical failure of instrumentation, including rod fracture, is closely linked to pseudarthrosis and high mechanical loads at stress-concentrated regions such as the lumbosacral junction. Multi-rod constructs have been introduced to improve construct durability in long fusions, and clinical series have demonstrated lower rates of rod fracture and pseudarthrosis at the lumbosacral junction when additional rods are used [5]. Despite such strategies, repeated mechanical failure may still occur in patients with poor biological fusion potential, as illustrated by this case.

Proximal junctional kyphosis and PJF are severe complications of long thoracolumbar constructs. PJF may include vertebral fracture, implant failure, or ligamentous disruption, potentially resulting in acute neurological deterioration. Known risk factors are advanced age, osteoporosis, fusion to the pelvis, and sagittal overcorrection [6, 7]. In the present case, junctional failure caused unstable thoracic fractures with neurological compromise, highlighting the potentially catastrophic nature of this pathology.

Beyond spinal mechanics, this case underscores the vulnerability of elderly patients undergoing repeated major spine surgery. Age-related medical complications, including delirium and cerebrovascular events, may delay or complicate surgical intervention and should be considered when planning revision strategies and balancing operative risk against neurological deterioration [8].

Overall, this case highlights the need for careful patient selection, optimization of bone health, appropriate fusion length, and precise sagittal alignment. In high-risk patients, long-term follow-up and multidisciplinary care are essential to limit progressive complications and preserve neurological function.
